# Methadone in Cancer-Related Neuropathic Pain: A Narrative Review

**DOI:** 10.3390/curroncol31120561

**Published:** 2024-12-01

**Authors:** Faten Ragaban, Om Purohit, Egidio Del Fabbro

**Affiliations:** Division of Palliative Medicine, Department of Internal Medicine, Medical College of Georgia, Augusta University, Augusta, GA 30912, USAopurohit@augusta.edu (O.P.)

**Keywords:** methadone, neuropathic cancer pain, cancer pain

## Abstract

**Background and Objective:** Cancer-related neuropathic pain (CRNP) is often a significant burden on patients’ quality of life. There are limited treatment guidelines for cancer-related neuropathic pain outside of CIPN. Although opioids are considered a third-line treatment option, no consensus exists on which opioid is most effective, either as a single agent or in combination with other medications. Our aim is to review and update the literature for methadone use in CRNP, since the last review was conducted in 2006. **Methods:** A comprehensive literature search was performed to evaluate the use of methadone in cancer-related neuropathic pain. Articles were identified from PubMed, Google Scholar, and Cochrane Library using the following keywords: “*methadone* AND *cancer pain* AND *neuropathic pain*” and “*cancer-related opioid treatment*”. **Results:** Studies were included if they evaluated methadone’s efficacy or safety in neuropathic pain management for patients with cancer. This review focused on randomized controlled trials (RCTs), systematic reviews, meta-analyses, and observational studies published between 2000 and 2024. Studies were excluded if they lacked specific data on cancer-related neuropathic pain or were case reports. **Conclusions**: The unique mechanisms of action and preliminary clinical trials support methadone’s status as the first opioid to consider for CRNP when non-opioid first-line treatments have failed to alleviate patient symptoms. Methadone can also be considered as a first-line opioid in patients with mixed nociceptive–neuropathic pain and any of the following features: renal dysfunction; administration of opioids through a feeding tube; a lack of financial resources/insurance; and a switch from another high-dose opioid. More research is needed regarding methadone for CRNP and methadone’s preferential use in specific sub-groups of patients.

## 1. Introduction and Background

Neuropathic pain presents as paresthesia, allodynia (pain from a stimulus that does not normally provoke pain), or hyperalgesia (increased sensitivity to a painful stimulus), and can be continuous or paroxysmal.

Causes of cancer-related neuropathic pain (CRNP) include chemotherapy-induced peripheral neuropathy (CIPN), radiation-related plexopathies, surgical procedures (post-mastectomy or post-thoracotomy), and direct tumor involvement of nerves. Comorbid related neuropathies (e.g., diabetic neuropathy) may coexist with CRNP and increase the risk of developing CIPN during chemotherapy. Additionally, patients may have both neuropathic- and nociceptive-type pain (mixed pain). A large systematic review of the prevalence of CRNP including over 13,000 patients noted an “estimate of 19% to 39.1% when patients with mixed pain were included” [1]. In clinical practice, it may be difficult to distinguish between the different causes and types of neuropathic pain (e.g., tumor vs. side effects of therapy) and the relative contributions of neuropathic vs. nociceptive pain in ‘mixed pain’ syndromes. Although the clinical presentation may prove challenging, the use of a detailed history and physical examination is the best approach to differentiating the type of pain patients are experiencing.

Both pharmacologic and non-pharmacologic interventions (e.g., exercise) are reported to alleviate CRNP. Our aim is to review the literature for methadone use in CRNP from 2000 to 2024 given that the last narrative review was published in 2006 [2]. There are a limited number of studies investigating methadone specifically in CRNP.

Current ASCO guidelines (2020) recommend duloxetine as the “only agent that has appropriate evidence to support its use for patients with established painful CIPN” [3]. The role of opioids as adjunctive therapy is not reviewed in this guideline. The European Society for Medical Oncology (ESMO) provides recommendations for CRNP, including “opioid combination therapies and carefully dosed adjuvants, when opioids alone provide insufficient pain relief” [4]. In general, opioids are not considered the first-line therapy for CRNP, and none of the guidelines recommend one specific opioid over another for CRNP. Although non-opioids are the first-line therapy, our review will focus on the potential superiority of methadone for CRNP compared to other opioids (either as a single agent or in combination with other medications).

### 1.1. Definitions and Diagnosis

A diagnosis of neuropathic pain is made clinically, based on patient history and the use of descriptive terms like “tingling, burning, shooting, numbness”. In 2020, ESMO outlined a diagnostic approach for CIPN, which includes both a history and a physical exam with an evaluation of sensory, motor, and autonomic components contributing to the sensation of pain [5]. CIPN can be a dose-limiting side effect, and in many cases, patients have residual effects even after treatment completion or discontinuation. There are validated methods for assessing chemotherapy-induced peripheral neuropathy. In a systematic review of 39 papers, the patient-reported outcome measures for CIPN with the most supporting evidence were the European Organization for Research and Treatment of Cancer Quality of Life Chemotherapy-Induced Peripheral Neuropathy Questionnaire (QLQ-CIPN20); and the Functional Assessment of Cancer Therapy/Gynecologic Oncology Group—Neurotoxicity (FACT/GOG-Ntx) [6].

Currently, there is no validated evaluation specifically for non-CIPN cancer-related neuropathic pain. This makes evaluation and further research challenging. For non-cancer-related neuropathic pain, the DN4 (Douleur Neuropathique en 4 Questions) or the International Association for the Study of Pain Special Interest Group on Neuropathic Pain (IASP-NeuPSIG) grading system has been used in studies. Both the DN4 and IASP-NeuSIG grading systems have been extrapolated for cancer-related neuropathic pain and, although used in some clinical trials, are seldom used in daily practice. One study showed that a higher rate of patients with CRNP were diagnosed using a clinician’s impression than using the DN4 questions or IASP-NeuSIG grading system, but there was closer concordance in diagnosis with the DN4 questions compared to the IASP-NeuSIG grading system [7].

Other tools include the Leeds Assessment of Neuropathic Symptoms and Signs (LANSS) and painDETECT (PDQ). They evaluate sensory items, clinical exam findings, and patient-reported symptoms. Similar to the DN4, they were developed for patients with non-cancer-related neuropathic pain. Given that these tools are more biased towards detecting sensory abnormalities, it may be difficult to adequately screen patients, since CRNP is often experienced in the presence of sensory loss [8]. A systematic review on the performance of screening tools in patients with CRNP found that LANSS and DN4 screening tools could generally distinguish between neuropathic and non-neuropathic pain in oncology patients, but with a decreased level of accuracy compared to non-cancer populations [9].

In clinical practice, a numerical rating scale is also frequently used to rate pain; however, it may not necessarily distinguish between neuropathic and other types of pain. The Edmonton Symptom Assessment Scale is a numerical rating system often used in daily practice (and symptom research) that surveys multiple symptoms including pain, anxiety, and depression. Further research on validated tools to diagnose cancer-related neuropathic pain is needed.

### 1.2. Methadone Mechanisms of Action

Methadone, an opioid agonist, is a potent synthetic analgesic with unique mechanisms of action. Developed in 1937 as an analgesic, it is often associated with medication-assisted therapy for patients with opioid use disorder.

Methadone is used for nociceptive and neuropathic cancer-related pain and can be administered through oral, rectal, intravenous, intramuscular, or epidural routes. Methadone has a large volume of distribution and is highly lipophilic, with a variable half-life of 7–59 h, much longer than its analgesic effect of 6–12 h. The long half-life can potentially lead to accumulation and adverse side effects if methadone is titrated upwards too quickly [10]. Methadone’s peak plasma concentration when given in tablet form is about 3 h, and it is metabolized in the liver by CYP 2B6 and to a lesser degree by CYP 3A4. Inhibitors of these enzymes such as fluconazole may increase methadone levels by as much as 35%, while inducers such as rifampin may reduce methadone levels and precipitate symptoms of withdrawal.

Compared to other opioids like morphine, methadone also acts as an agonist of κ- and σ-opioid receptors, as an antagonist of the N-methyl-D-aspartate (NMDA) receptor, and as an inhibitor of serotonin and norepinephrine uptake [10]. NMDA antagonism is thought to be the primary mechanism in alleviating neuropathic pain, central sensitization, and hyperalgesia by modulating pain stimuli propagation. Glutamate and Substance P play a major role in activating NMDA receptors [11,12,13]. Glutamate released from cancer cells may induce hyperalgesia by potentially activating peripherally expressed NMDA receptors [11,14]. The noradrenergic system and serotonin also play a role in neuropathic pain by modulating descending, pain-inhibitory pathways [15].

## 2. Methods

### 2.1. Search Strategy

**Figure 1:**
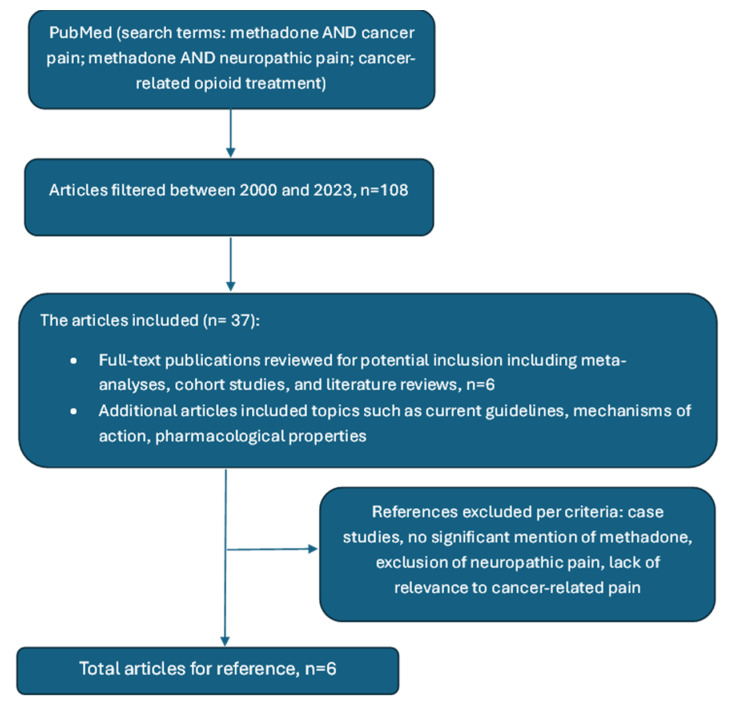
Schema of literature review.

A narrative review was conducted to evaluate methadone’s role as a first-line opioid for managing cancer-related neuropathic pain. A structured literature search was performed using PubMed (Figure 1). The following keywords were employed:

- “Methadone AND cancer pain”;
- “Methadone AND neuropathic pain”;
- “Methadone AND chemotherapy-induced peripheral neuropathy”;
- “Cancer-related opioid treatment”.

The search included studies published between 2000 and 2024.

### 2.2. Inclusion and Exclusion Criteria

Studies were included if they

- Were published in English;
- Focused specifically on methadone as a treatment for cancer-related neuropathic pain;
- Investigated methadone’s analgesic effects either alone or in comparison with other opioids;
- Featured randomized controlled trials (RCTs), systematic reviews, meta-analyses, observational studies, and guidelines.

Studies were excluded if they

○ Addressed only non-cancer-related neuropathic pain;
○ Were case series or reports lacking robust methodology;
○ Focused on non-opioid therapies for neuropathic pain.

### 2.3. Analysis and Synthesis

The narrative synthesis focused on identifying methadone’s clinical advantages that support its role as the first-line opioid for neuropathic pain. The methodologies of clinical studies were identified and findings were contextualized within existing opioid treatment frameworks for cancer-related neuropathic pain management.

## 3. Results

The following studies report the efficacy and safety of methadone, either as a single agent or in combination therapy at relatively low doses, for cancer-related neuropathic pain. Brief summaries of the included studies are shown in Table 1.

### 3.1. Clinical Studies of Methadone for Cancer-Related Neuropathic Pain

There are few randomized controlled trials with methadone in cancer-related neuropathic pain, and recruitment to studies is challenging [21]. There are, however, preliminary RCTs and retrospective studies indicating that methadone could be considered the opioid of choice in CRNP and in oncology patients with mixed nociceptive–neuropathic pain.

#### 3.1.1. Randomized Controlled Trials

A randomized trial evaluated 74 women aged 18–70 years old diagnosed with stage II-III cervical cancer experiencing neuropathic pain. The study compared the efficacy and safety of methadone versus immediate-release (IR) morphine over a 12-week period. Inclusion criteria included pain severity > 5/10 evaluated by a numeric rating scale (NRS) and neuropathic pain diagnosis based on a Douleur Neuropathique 4 (DN4) score > 4. The methadone group received doses of 2.5–20 mg/day, while the IR morphine group received 30–360 mg/day. Secondary outcomes included opioid-related side effects, the need for co-analgesics, and overall treatment tolerability. Methadone provided more rapid and effective pain relief and fewer side effects compared to IR morphine. The final NRS pain score was lower in the methadone group (1.5) compared to the morphine group (2.7). Of note, co-analgesic medications (sodium valproate or NSAIDs) were allowed to be added if clinically indicated for both the morphine and methadone arms. Methadone was also superior in reducing DN4 scores, with many patients in the methadone group reporting no detectable neuropathic pain by the study’s conclusion. Also, methadone’s use in a low-resource setting has additional benefits given that methadone is inexpensive compared to other long-acting opioids. Limitations of this study include the use of IR morphine rather than a slow-release formulation and potential biases due to the single-blind design [16].

A single-center, open-label, randomized controlled trial compared methadone (4–10 mg/day) to transdermal fentanyl (12–25 mcg/h) in patients with CRNP and head and neck cancer. A total of 52 participants, naive to strong opioids, with baseline pain > 4 on an NRS and a neuropathic pain component (DN4 score of >4) were included, as well as some patients with mixed nociceptive–neuropathic type pain. Methadone was more effective than TD fentanyl in reducing pain at both 1 week and 3 weeks (*p* = 0.0421). Clinical success was achieved faster in the methadone group, with 50% reporting a 50% pain reduction at 1 week, compared to 15% in the fentanyl group (*p* = 0.012). The difference in pain reduction between the two groups was significant at 1 and 3 weeks but not at 5 weeks. Side effects were similar between groups, though methadone showed a trend towards better patient-reported global perceived effects, which the authors cite as the “patient’s belief about the efficacy of treatment”. Limitations included the relatively small sample size and a significant loss to follow-up by the end of 5 weeks (about 50% of patients from both the TD fentanyl and methadone groups). Additionally, the open-label design may have introduced bias, and the study was underpowered to detect differences in long-term outcomes or side effects [17].

#### 3.1.2. Retrospective Studies

Methadone may help reduce pill burden and the potential complications of adjuvant pain medications with opioids. One retrospective, observational study from Japan included patients with genitourinary, GI, breast, lung, and head and neck cancers who were experiencing CRNP. Of note, these patients were previously on other opioids for an average of 321 days but experienced inadequate pain relief or intolerable side effects prior to switching to methadone. Investigators reported that 78.6% of patients who were successfully switched to methadone experienced significant pain relief. The mean FACES Pain Scale score was reduced from 4.43 to 1.86 within 14 days. Moreover, switching to methadone reduced or eliminated the need for adjuvant analgesics such as pregabalin, corticosteroids, tramadol, and duloxetine in 70.5% of patients. However, since only 28 patients enrolled and the study lacked a control group, the generalizability of the findings is uncertain. Moreover, the use of the FACES Pain Scale, typically used for pediatric and older adult patients, may not have been the most appropriate tool for the middle-aged adult population in this study [18]. Another chart review of 31 patients with CIPN on a variety of opioids, anticonvulsants, and anti-depressants found that 65% of patients perceived methadone to be effective in relieving their neuropathic pain [19].

A retrospective cohort study evaluated 274 patients with spinal metastasis from a variety of cancers, experiencing mixed nociceptive pain and neuropathic symptoms, specifically numbness. Patients were treated with either methadone, tapentadol, hydromorphone, oxycodone, or fentanyl. Pain severity was evaluated with numerical pain scores. In patients without numbness, there was no significant difference observed after 1 week in terms of numerical pain score reduction in the methadone group compared to the other opioids. However, in patients who had pain with numbness, methadone was significantly more effective in reducing pain. Additionally, in the group of patients who switched to methadone, the total daily morphine equivalents prior to switching was significantly higher (~95 mg/day) than the other opioid study groups. The authors hypothesized that patients may have been experiencing opioid-induced hyperalgesia and that a switch to methadone reduced pain scores via NMDA-receptor antagonism [11].

Given the efficacy of methadone and duloxetine for CRNP and CIPN, respectively, a retrospective study of 43 patients in a supportive care clinic hypothesized that a combination of the medications would be better than monotherapy alone [20]. Three patients reported neuropathic pain, while the rest reported mixed nociceptive and neuropathic cancer-related pain. Edmonton Symptom Assessment Scale (ESAS) scores were compared before and after combination therapy was initiated. Pain and anxiety scores decreased significantly with combination therapy, by 0.9 (SD = 3.0, *p* = 0.052) and 1.1 (SD = 2.8, *p* = 0.018), respectively, and 28% of patients had a two-point reduction in pain scores, while 30% had a one-point decrease. The high adherence rates (81%) were in contrast to another study of methadone–duloxetine in HIV patients with polyneuropathy, in which adverse effects were frequent, associated with high rates of study dropout, and precluded trial completion. The authors of the study postulated that high-dose methadone (30 mg daily, in divided doses by day 11) and the opioid-naïve status of participants may have contributed. In contrast, oncology patients tolerated combination therapy for at least 4 weeks, with median methadone doses of 15–30 mg daily [20]. This preliminary observational study was limited to a single National Cancer Institute (NCI) cancer center; however, the relatively few side effects and the acceptability over a sustained period in patients with cancer suggest that prospective RCTs are warranted.

## 4. Discussion

Based on the studies identified in our literature search, methadone could be considered as a first-line opioid choice for CRNP in patients for whom non-opioid therapies fail to provide adequate relief. However, it may be challenging to categorize an individual patient’s pain experience into pure neuropathic or nociceptive pain. Approximately 20% of patients with cancer pain have mixed-type pain (both neuropathic and nociceptive), while about 60% experience pure nociceptive pain [22]. A meta-analysis of over 10,000 patients reported a prevalence of about 40% for either pure neuropathic or mixed pain [1]. Since there is evidence showing its non-inferiority to other opioids for nociceptive pain, methadone could also be considered as a preferred opioid when uncertainty exists with regard to categorizing cancer-related pain. For example, a clinician suspecting that a neuropathic component may coexist with a patient’s predominantly nociceptive pain complaint could reasonably choose methadone as the preferred opioid.

There are other populations where methadone may be considered preferentially as a first-line opioid.

**Figure 2:**
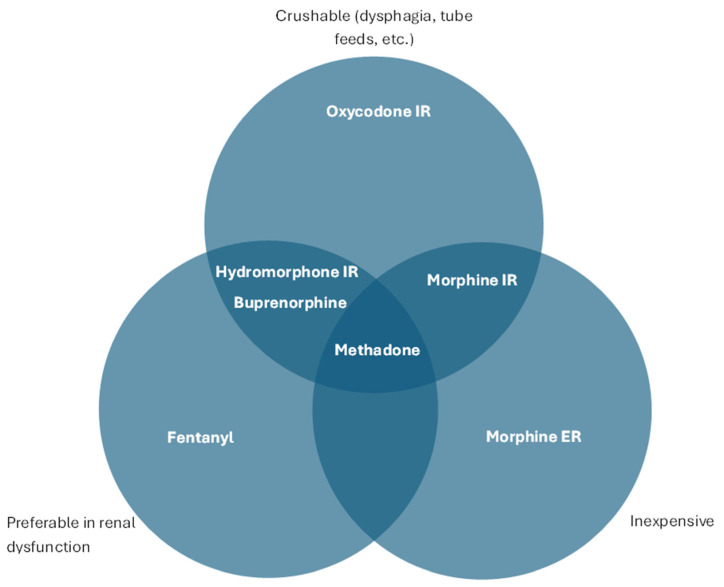
Comparison of opioid properties and clinical roles.

Besides cancer-related neuropathic pain, methadone has additional benefits in specific populations of patients with cancer, including patients with renal dysfunction; patients with feeding tubes who require long-acting opioids; and patients with limited financial resources or who require opioid rotation/switching from a high-dose opioid (Figure 2).

In patients with kidney dysfunction, methadone does not accumulate and is minimally removed by hemodialysis, supporting its preferential use as an analgesic in this group of patients. Methadone is excreted in the gastrointestinal tract, preventing the accumulation of the drug and any active metabolites. This is in contrast to other opioids such as morphine or oxycodone, which are predominantly renally cleared, thereby increasing the risk of metabolite accumulation and side effects. Similarly, in patients on dialysis, methadone is the preferred opioid because it is minimally removed with dialysis, so that levels and analgesia are less likely to fluctuate [12,23]. Fentanyl, buprenorphine, and, to a lesser degree, hydromorphone may also be safer to use in patients with renal dysfunction compared to morphine, because of accumulation of metabolites.

In patients who are unable to take whole pills by mouth, their options for long-acting opioid medications are limited, given that extended-release medications should not be crushed. Methadone maintains its long-acting analgesic properties even when pills are crushed or in liquid form. Transdermal fentanyl may not be ideal in patients with severe cachexia or night sweats, and frequent four-hourly administration of immediate-release opioids may be too burdensome for patients and families. Methadone can be crushed or administered in liquid form through a feeding tube without affecting its extended-release analgesic properties.

Compared to all other long-acting opioids, methadone is relatively less expensive and should be considered for patients with limited financial resources or in resource-poor regions (if available). U.S. pricing for 5 mg methadone may be as low as USD 0.07 per tablet [24]. Despite the low cost, methadone appears to be superior to other opioids in a number of clinical contexts described in this review. The stigma attached to methadone because of its use for addiction therapy may be a hindrance to wider acceptance of its use.

Methadone’s efficacy and safety in pediatric oncology patients is gathering increasing evidence, although this population is beyond the scope of our review. Some retrospective studies show a significant pain decrease with no arrythmias or neurotoxicity in children with advanced cancer [25,26].

Patients with CRNP and a history of substance use disorders, specifically opioid use disorders (OUDs), are often challenging to manage. Methadone and buprenorphine-naloxone are the only two FDA-approved medications for the treatment of OUD. A white paper from 2016 on the use of methadone in hospice and palliative care recommended avoiding methadone in patients with an active substance use disorder, citing a Canadian case–control study that found an increased risk of overdose in patients, specifically those using benzodiazepines or antipsychotics in combination with methadone [27].

Although there are no specific studies evaluating the comparative safety of opioids in palliative care patients with a history of OUD, recent evidence has emerged showing that methadone for OUD may have better adherence rates without an increased mortality risk when compared to buprenorphine–naloxone [28]. The study, a population-based retrospective cohort study using linked administrative health databases, was conducted in response to guidelines from British Columbia explicitly endorsing buprenorphine–naloxone as the first-line treatment. Previously, a systematic review (2023) including RCTs and observational studies also reported a better retention rate for methadone compared to buprenorphine among patients treated for OUD. The U.S Substance Abuse and Mental Health Services Administration does not explicitly endorse one intervention over another, although only buprenorphine–naloxone is available in both specialized drug treatment centers and office-based settings (methadone is not available). Given the potential benefit of mitigating risk in oncology patients with a history of SUD and the inconsistent guidelines regarding methadone’s preferential use in OUD, there is a clear need for more research in this area.

Other opioids, including levorphanol and buprenorphine, are reported to be effective for cancer-related neuropathic pain; however, the evidence is more limited compared to that for methadone. Most studies are limited to case reports or for the relief of nociceptive cancer-related pain [29]. Some of buprenorphine’s potential advantages include a decreased risk of respiratory depression, a lower risk of decreasing testosterone levels in men, no risk of serotonin syndrome in combination with antidepressants, and multiple options for administration, given the transdermal, trans-mucosal, and sublingual formulations [30]. In addition, buprenorphine shares some of the advantages of methadone in patients with kidney failure [31] or substance use disorder (SUD). However, methadone is far less expensive and can be administered via a number of routes including intravenous, subcutaneous, oral, feeding tube, or rectal. A recent systematic review and network meta-analysis included 16 RCTs comparing strong opioids for the treatment of cancer-related pain (not against no intervention, weak opioids, or placebo). The authors suggest that methadone deserves “further promotion as a first-line treatment for moderate to severe cancer-related pain” given the evidence for a higher likelihood of success after one week of therapy [32].

### Limitations of Methadone

Methadone has side effects common to other opioids, including some degree of constipation, risks of respiratory depression, or drowsiness. A risk of overdose is also possible, especially when given in combination with benzodiazepines, alcohol, or other CNS depressants. Patients need to be informed about the risks, emphasizing the need to safely store medications and take them as prescribed. Polysubstance use disorders are very prevalent, and clinicians should be especially careful to avoid prescribing combination benzodiazepines and opioids, particularly methadone. An opioid maintenance program reported higher doses (>120 mg daily) of methadone combined with benzodiazepines increased the risk of overdose [33]. Given that gabapentinoids are also commonly prescribed for neuropathic pain, clinicians must be mindful of the increased risk of adverse events, including respiratory depression, sedation, and overdose, when used in combination with opioids. A large observational study among patients receiving prescription opioids found that concomitant treatment with gabapentin was associated with a substantial increase in the risk of opioid-related death. Moderate- or high-dose gabapentin use (≥900 mg daily) was associated with an increase of almost 60% in the odds of opioid-associated death [34].

Clinicians should be mindful of the interactions between methadone and medications metabolized by CytP450, particularly in patients with impaired liver function [12]. Additionally, there is wide inter-individual variation in the metabolism of methadone and uncertainty with regard to equianalgesic doses when switching from other opioids.

Generally, as a first-line opioid, doses should start low (2.5 mg q12 h to q6 h PO) and escalate slowly given methadone’s long half-life and that it can take up to 5 days or more to reach a steady state. The methadone–morphine equianalgesic dose may be 1:10 or even 1:20, especially at higher doses of morphine, and a ceiling dose of 30 mg methadone per day in divided doses should be considered as a starting point when switching from a MEDD > 300 mg [35]. While caution with methadone dose escalation is essential, it should be noted that a ‘German model’ using the more potent Levo-isomer of methadone, allowed for 5 mg *as-needed* dosing (in most countries, only racemic methadone is available). Almost all the patients included in a retrospective study had advanced cancer, and about half experienced neuropathic pain. The authors suggest that as-needed dosing is better suited for responding to inter-individual variation and reported that conversion to *Levo*-methadone was accomplished with no serious adverse effects [36].

QTc prolongation is a potential side effect of methadone, although it appears to be dose-dependent and more frequently encountered in patients on high doses (65–1000 mg/day) [12]. The consensus white paper on the use of methadone in hospice and palliative care outlines some of the precautions regarding EKG monitoring depending on prognosis, and methadone dose escalation [27]. However, applying QTc monitoring guidelines stringently in the palliative care setting may be a barrier to adequate pain control [37].

A critical question is whether QTc prolongation is clinically significant. A prospective study of 132 patients with opioid use disorder (without cancer) reported that QTc intervals increased by a mean of 10.8 milliseconds from baseline to follow-up (*p* < 0.001). Patients in the highest tertile of methadone (110–150 mg) experienced a mean increase of 13.2 msec. No episodes of Torsades de pointes were observed or reported during the study period, and no one exhibited a QTc interval of greater than 40 milliseconds [38]. Similarly, in patients with cancer, a sample of 26 patients in an ambulatory setting showed no prolongation of the QTc interval on a median dose of 30 mg methadone after 3 months [39].

Since clinicians generally avoid the combination of methadone and ondansetron because of concern surrounding QTc prolongation, alternative anti-emetics such as metoclopramide and olanzapine may be preferable especially for non-CINV, although these agents are also reported to increase the risk of prolonged QTC to a variable degree.

For cancer-related *nociceptive* pain, some studies have reported the superiority of methadone as a first-line treatment in terms of greater efficacy [40] and fewer adverse effects [41], while others, including a RCT comparing methadone to morphine, reported no superiority [42]. Although the studies showing the superiority of methadone are inconsistent in nociceptive pain, methadone is clearly an effective analgesic, and factors other than neuropathic pain may favor methadone as the preferential opioid (Figure 2).

## 5. Conclusions

We acknowledge that opioids are not first-line therapy for CRNP, but if opioids are needed for symptom management, methadone might be a more effective choice than other opioids. The unique mechanisms of action and preliminary clinical trials provide support for methadone’s use as a potential opioid of choice for CRNP. Methadone could also be considered as a first-line opioid in patients with mixed nociceptive–neuropathic pain and any of the following features: renal dysfunction; administration of opioids through a feeding tube; a lack of financial resources/insurance; and a switch from another high-dose opioid. Best practices for the diagnosis and assessment of CRNP is an area that needs further research. Given the limited high-quality studies, more research is needed regarding methadone for CRNP and methadone’s preferential use in specific sub-groups of patients.

## Figures and Tables

**Table 1:** Summary of included studies and findings.

Study Authors and Date	Sample Size/Description	Methodology	Key Outcomes
Adumala A. Oral Methadone versus Morphine IR for Patients with Cervical Cancer and Neuropathic Pain: A Prospective Randomized Controlled Trial. (2023) [16]	74 cervical cancer patients with neuropathic pain, over a 6-month period.	Prospective randomized controlled trial.	Methadone provided superior pain relief compared to morphine IR, with fewer opioid-related side effects.
Haumann, J. Methadone Is Superior to Fentanyl in Treating Neuropathic Pain in Patients with Head-And-Neck Cancer. (2016) [17]	52 head and neck cancer patients with neuropathic pain, over a 12-week period.	Prospective randomized controlled trial.	Methadone was superior to fentanyl in managing neuropathic pain, with better pain relief and fewer dose escalations.
Sugiyama, Y. A Retrospective Study on the Effectiveness of Switching to Oral Methadone for Relieving Severe Cancer-Related Neuropathic Pain and Limiting Adjuvant Analgesic Use in Japan. (2016) [18]	28 patients with severe cancer-related neuropathic pain, over a 2-year retrospective period.	Retrospective cohort study.	Switching to oral methadone significantly relieved neuropathic pain and reduced the need for adjuvant analgesics.
Boen, C. Methadone for Pain Management in Chemotherapy-Induced Peripheral Neuropathy: A Retrospective Review.(2024) [19]	31 cancer patients with chemotherapy-induced peripheral neuropathy, over a 2-year period.	Retrospective cohort study.	Methadone reduced pain intensity in 78% of patients; decreased need to address breakthrough pain in65%; and had minimal side effects.
Curry, Z.A. Combination Therapy with Methadone and Duloxetine for Cancer-Related Pain: A Retrospective Study. (2021) [20]	43 cancer patients with neuropathic pain, over a 1-year retrospective review period.	Retrospective cohort study.	Combination therapy of methadone and duloxetine provided superior pain relief and reduced opioid consumption in 73% ofpatients.
Takemura, M. Differences in the Analgesic Effect of Opioids on Pain in Cancer Patients with Spinal Metastases. (2023) [11]	274 cancer patients with spinal metastases and severe pain, over a 1-year period.	Retrospective cohort study.	Methadone showed superior pain control compared to other opioids in patients with spinal metastases, particularly for neuropathic pain.
